# Umweltfürsorge im Krankenhaus: Hygienische Sauberkeit und die feminisierte Arbeit an der Atmosphäre

**DOI:** 10.1007/s00048-020-00289-x

**Published:** 2020-12-17

**Authors:** Käthe von Bose

**Affiliations:** Campus Griebnitzsee, Universität Potsdam, August-Bebel-Str. 89, 14482 Potsdam, Deutschland

**Keywords:** Krankenhaus, Hygiene, Geschlecht, Atmosphäre, Umwelt, Hospital, Hygiene, Gender, Atmosphere, Environment

## Abstract

Den Boden putzen, das Bett abziehen, einen Blumenstrauß arrangieren – Bemühungen um Sauberkeit sowie eine angenehme Raumatmosphäre obliegen im Krankenhaus meist weiblichen* Pflegerinnen, Reinigungskräften und Hauswirtschafterinnen. Im Klinikalltag vermischen sich Anforderungen an hygienische Sauberkeit unter Prozessen der Ökonomisierung mit Logiken des Marketings sowie mit affektiv-emotionalen Bedürfnissen der Akteur_innen dieser Räume. Obwohl die Maßstäbe klinischer Hygiene auf medizinischem Wissen basieren, sind die Arbeitsteilung sowie Ansprüche an Sauberkeit auf verschiedenen Hierarchieebenen zugleich von vergeschlechtlichten und teils rassifizierten Vorstellungen durchdrungen, die über den klinischen Kontext hinausweisen. Dies legt schon eine Beschäftigung mit der Geschichte der Bakteriologie nahe: Die Logik und Sprache der Infektionsabwehr ist in Wissenschaft und Alltag auch verwoben mit sozialen Differenzmarkierungen.

Unter Rückgriff auf die Ergebnisse einer Ethnografie zu Sauberkeit und Reinigungsarbeiten im Krankenhaus, die wissensgeschichtlich fundiert werden, wird in dem Beitrag die Frage nach der (feminisierten) Sorge für die Umwelt mit der Frage nach der Atmosphäre klinischer Räume verknüpft. Auf welche Weise und mit welchen Effekten verschränken sich wissenschaftlich-medizinisches Hygienewissen mit einem alltäglichen, jedoch historisierbaren Wissen über schöne und angenehme Sauberkeit, das immer noch weiblich konnotiert ist?

## Abstract (en)

Cleaning the floor, stripping the bed, arranging a bouquet of flowers—such tasks are essential to keeping a hospital room clean and creating a pleasant atmosphere. They usually fall under the purview of female* nurses, cleaning staff and housekeepers. In everyday hospital life, the demands for hygienic cleanliness commingle with the imperatives of economization, marketing logic, and attention to the affective and emotional needs of the actors in these rooms. Although the standards of clinical hygiene are based on medical knowledge, the division of labor and the demands for cleanliness at various hierarchical levels also reveal gendered and partly racialized ideas that point beyond the clinical context. This blending of imperatives in the hospital environment invites deeper consideration of the history of bacteriology: The logic and language of defense against infection in science and everyday life is also interwoven with social markers of difference.

Drawing on the findings of an ethnography on cleanliness and cleaning work in hospitals, as well as a history of knowledge approach, the article links the question of (feminized) care for the environment with the question of the atmosphere of clinical rooms. In what ways, and to what effect, does scientific knowledge about medical hygiene also carry with it cultural and aesthetic perceptions of beautiful and pleasant cleanliness that reveal feminine connotations rooted in the nineteenth century?

Fällt der Begriff der Atmosphäre im Zusammenhang mit Krankenhäusern, ist damit meist eine Abwertung verbunden. Im Kontext von architektonischen Umgestaltungen etwa soll die Krankenhausatmosphäre durch ein „Wohlfühl-Ambiente“ (Kunststylist 2020) oder durch Hotelkomfort ersetzt werden. Und umgekehrt: Wird in Reiseberichten ein Hotel als besonders wenig wohnlich beschrieben, findet sich zuweilen die gleiche Assoziation (Tripadvisor 2020). Die „typische Krankenhaus-Flur-Atmosphäre“ (Mewis 2017) soll möglichst überall vermieden werden. Was damit gemeint ist, scheint kaum einer Erklärung zu bedürfen, gehören die Assoziationen, die der Begriff aufwirft, doch zum kulturellen Wissen. Krankenhausatmosphäre bezeichnet das typische Zusammenspiel aus weißen, höchstens karg dekorierten Wänden, funktionaler Einrichtung, medizinischen Geräten und dem unverkennbaren Geruch nach Desinfektionsmitteln. Die medizinisch positiv konnotierte Sterilität kippt im Alltagsdiskurs über Krankenhäuser in das im Begriff ‚steril‘ enthaltene Gegenbild: Gefühlskälte, mangelnde Empathie, Unnatürlichkeit.

Was unter Krankenhausatmosphäre verstanden wird, ist trotz seiner scheinbaren Universalität nichts, was von sich aus und ein für alle Mal besteht. Selbst ein so wenig greifbares Phänomen wie die Atmosphäre eines Raumes hat zum einen eine Geschichte, zum anderen entsteht es auf gewisse Weise immer wieder neu. Die Atmosphäre klinischer Räume ist zurückzuführen auf die architektonische Gestaltung der Räume, die wiederum auf dem medizinischen und speziell krankenhaushygienischen Wissen seiner Zeit fußt, das nach bestimmten Strukturen verlangt und sich stets weiterentwickelt oder erneuert. Sie basiert auf politischen Entscheidungen ebenso wie auf der alltäglichen Arbeit unterschiedlichster Akteur_innen, die sie aufrechterhalten, ausfüllen, verändern.

Die Arbeit an einer hygienisch reinen und zugleich angenehmen Raumatmosphäre ist historisch weiblich konnotiert. Unter hygienischer Reinheit wurde dabei nicht immer nur Krankheitsprävention verstanden, sondern es waren auch ästhetische Gesichtspunkte damit verbunden. So arbeitet Susanne Breuss für die Hygienebewegung des 19. Jahrhunderts heraus, dass hygienische Sauberkeit im Privathaushalt auch „mit Schönheit gleichgesetzt“ wurde und „für gute Laune und eine angenehme Atmosphäre sorgen“ (2006: 116) sollte. Zuständig für das Herstellen dieser Atmosphäre war die moderne Hausfrau, die laut Breuss mit dem Hygienediskurs erst konstituiert wurde (ebd.). Wie auch in Kira Jürjens Beitrag in diesem Heft deutlich wird, umfasste das Aufrechterhalten dieser häuslichen Umwelt eine Fülle textiler, reinigender Praktiken. Das Schaffen einer angenehmen Atmosphäre mit den Mitteln der Reinigung und des Ordnens ist historisch auch schon deshalb eine feminisierte Tätigkeit, weil es sich auf die Sphäre des Privaten, des Haushalts und der Familie bezieht.

In heutigen Krankenhausräumen bestimmen Sauberkeit, Hygienewissen und die Routinen der Krankenhaushygiene die räumliche Atmosphäre maßgeblich mit. Dies wird bereits angesichts der Assoziation von Sterilität deutlich, die in dem Begriff Krankenhausatmosphäre enthalten ist. Inwiefern Mikroorganismen als unbedingte Krankheitsauslöser zu verstehen sind oder Erreger ihre Virulenz – ihre Fähigkeit, eine Krankheit hervorzurufen, – erst im Zusammenwirken mit dem Wirt und der Umwelt, dem Milieu, erhielten, stellte in der Medizingeschichte eine wegweisende Debatte dar. Historisch ist diese besonders im Kontext der Wende von Hygienebewegung zu Bakteriologie zu verorten (Sarasin 2007; Mendelsohn 2002; Schlich 1996). In diesem Beitrag geht es zwar weniger um die medizinische Konzeption von Umwelt als Einflussfaktor von Krankheiten als vielmehr um die Frage, wer in klinischen Räumen die Umwelt der zu behandelnden und zu versorgenden Patient_innen bearbeitet und sie damit als Möglichkeitsraum erst herstellt. Medizinische Debatten bestimmen diese Arbeit jedoch stets mit und bilden den Hintergrund der Aushandlungen um die Arbeit an der Umwelt und räumlichen Atmosphäre.

Diese Arbeit umfasst unterschiedliche Praktiken. Reinigung und Desinfektion gehören ebenso dazu wie das Aufräumen von Warteräumen bis hin zu Dekorationen etwa zur Weihnachtszeit, die der (gefühlten) Sterilität klinischer Räume entgegenwirken sollen. Diese – formal zugewiesenen oder informell verteilten – Aufgaben obliegen je nach Personalstruktur des jeweiligen Krankenhauses Pfleger_innen, Reinigungskräften oder Hauswirtschafter_innen. Dabei handelt es sich überwiegend um Berufe und Arbeitsbereiche, die nach wie vor vergeschlechtlicht sowie teilweise ethnisiert und rassifiziert sind und häufig unter prekären Arbeitsbedingungen verrichtet werden (von Bose 2017). Das *Cocooning*, das in diesem Beitrag analysiert wird, ist damit insofern ein spezifisches, als es im Kontext von Erwerbsarbeit in der Institution Krankenhaus verortet ist und sowohl in formalisierte Zuständigkeiten, als auch in informelle Praktiken eingebettet ist, die auf je unterschiedliche Weise vergeschlechtlicht sind.

Unter Rückgriff auf die Ergebnisse einer Ethnografie zu Sauberkeit und Reinigungsarbeiten im Krankenhaus (von Bose 2017) wird in dem Beitrag die Frage nach der (feminisierten) Sorge für die Umwelt mit der Frage nach der Atmosphäre klinischer Räume verknüpft, die in dem Forschungsfeld wiederholt thematisiert wurde (ebd.: 133–134, 142–145; von Bose 2019a). Von welchen Wissensordnungen ist die Arbeit an einer guten, vertrauenerweckenden Atmosphäre in diesem Kontext geprägt? Auf welche Weise und mit welchen Effekten verschränken sich wissenschaftlich-medizinisches Hygienewissen mit einem alltäglichen, jedoch historisierbaren Wissen über schöne und angenehme Sauberkeit, das immer noch weiblich konnotiert ist?

Für die zugrundeliegende Studie (von Bose 2017) habe ich zum einen in zwei deutschen Universitätskliniken im Zeitraum von 2010 bis 2014 teilnehmend beobachtet, insbesondere in den Bereichen Reinigung, Hauswirtschaft und Pflege. Zum anderen habe ich 13 narrative, leitfadengestützte Interviews sowie zahlreiche ethnografische Gespräche mit Akteur_innen dieser Arbeitsbereiche geführt sowie unter anderem auch mit Mitarbeiter_innen der Sterilisation, mit Ärzt_innen, Patient_innen und Besucher_innen. Methodisch habe ich mit dem Forschungsstil und den Analysewerkzeugen der Grounded Theory (Strübing 2008; Strauss & Corbin 1996) gearbeitet.

Der vorliegende Beitrag verbindet die ethnografische Untersuchung konkreter Herstellungsprozesse von Umwelt und Atmosphäre mit einem wissensgeschichtlichen Zugang. Letzterer stand bei der Konzeption des Forschungsdesigns der Ethnografie zwar nicht im Vordergrund, die Verweise auf medizin- und geschlechtergeschichtliche Kontinuitäten verleihen den zeitgenössischen Daten jedoch eine historische Tiefenschärfe und ermöglichen eine fundiertere Analyse. Dafür gehe ich zunächst auf zentrale Begriffe und historische Entwicklungen ein, die grundlegend sind für die daran anschließende Analyse des empirischen Materials. Für deren Darstellung habe ich eine räumliche Struktur gewählt: In einer Bewegung vom Krankenbett über das Krankenzimmer bis zur Station wird es darum gehen, der Feminisierung der Arbeit an der räumlichen Umwelt und Atmosphäre in ihren je spezifischen Ausformungen nachzugehen. Dabei wird zum einen deutlich werden, dass es bei diesen Arbeiten und ihrer Aushandlung nicht nur um Geschlecht, sondern auch um Differenzsetzungen entlang von Klasse und Rassifizierung geht. Zum anderen wird evident, wie grundlegend die Arbeit an der Umwelt ist und wie sie in das soziale Gefüge von Krankenhausräumen eingebettet ist.

## Grundlegendes: Hygienediskurse, Umweltfürsorge, Atmosphäre

Schon zu Beginn der Entstehung von Krankenhäusern im 18. Jahrhundert galt „das Hauptaugenmerk bei der Einrichtung dieser neuen Institutionen der Sauberhaltung“ (Murken 1990: 2); das Krankenhaus wurde gar zum „Paradigma der Sauberkeitsausbreitung“ (Mönkemeyer 1988: 186). Hygienische Bestimmungen und Praktiken der Reinigung in Krankenhäusern unterlagen jedoch steten Wandlungsprozessen. Worauf bei der Sauberhaltung geachtet werden sollte und was unter Sauberkeit verstanden wurde, änderte sich über die Zeit.^1^

Vor den Erkenntnissen der Bakteriologie, die die klinische Hygiene und Regularien der Reinigung auf das Bekämpfen von Erregern konzentrierte, achtete man dem Krankenhaushistoriker Axel Murken zufolge in der Planung und Errichtung von Krankenhäusern schon auf die „Reinhaltung der Luft, die Sauberkeit der Innenräume und die schnelle Abfall- und Abwasserbeseitigung“ (Murken 1990: 5). Angenommenen Gefahren etwa durch Ausdünstungen von Patient_innen konnte durch die fortschreitenden technischen Errungenschaften nachgekommen werden (ebd.). Die Bakteriologie wiederum, deren Erkenntnisse gegen Ende des 19. Jahrhunderts einen Einschnitt in bisherige hygienische Theorien und Konzepte darstellten, ist für die Geschichte der Medizin von unbestrittener Bedeutung (Sarasin et al. 2007: 15). War zwar das Konzept von Krankheitsübertragung durch unsichtbare Stoffe nicht neu, so ermöglichte die Bakteriologie eine Aufdeckung der Ursachen sowie der Wege einer Übertragung sehr viel genauer. Durch Isolation, Desinfektion, später auch Immunisierung konnte nun systematisch und gezielt gegen Krankheitserreger vorgegangen werden (Labisch 1992: 132). Die bakteriologischen Erkenntnisse bestimmten daraufhin sowohl die alltäglichen Hygienepraktiken (Vigarello 1992) als auch die Krankenhausarchitektur; so wurde ab den 1880er Jahren neben neueren Technologien wie Lüftungsanlagen etwa mehr auf die Möglichkeiten einer einfachen Reinigung von Gegenständen und Entsorgung von Abfällen geachtet (Murken 1990: 19). Auch die von Robert Kochs Erkenntnissen geleiteten Desinfektions- und Sterilisationsapparate wurden immer selbstverständlicher. Von den Materialien und Gegenständen weiteten sich nun etwa Desinfektionsmaßnahmen auch auf die Patient_innen und das Personal aus (Labisch 1992). Thomas Schlich betont mit Charles Rosenberg, dass keine wissenschaftliche Erkenntnis zu solch grundlegenden Umgestaltungsprozessen im Krankenhaus geführt habe wie die „germ theory“ (Schlich 2007: 237). Einer der Gründe für die Überzeugungskraft wird in der Sichtbarmachung der Keime gesehen: In Laboren konnte mit neuen Technologien (Mikroskopen, Nährflüssigkeiten, Färbemitteln) sichtbar gemacht werden, was bisher unsichtbar gewesen war und auch weiterhin für das bloße Auge und damit im Alltag unsichtbar blieb (Sarasin 2007: 434).

Dieses Changieren zwischen Sichtbarkeit und Unsichtbarkeit bleibt bis heute ein Thema aller Tätigkeiten, die hygienische Reinheit im Sinne der Infektionsabwehr herstellen soll – nicht nur, aber eben auch in Krankenhausräumen. So lassen sich Kontinuitäten des Hygienewissen aus dem 19. Jahrhundert auch in zeitgenössischen Diskursen nachvollziehen. Hygienewissen, speziell das Wissen über Infektionsabwehr, Prävention und Gesundheit verfügt dabei neben einer medizinischen und mikrobiologischen stets auch über eine soziale Dimension, schon insofern als es alltäglich ausgeformt, eingeübt und ausgehandelt werden muss. Dies lässt sich aktuell im Kontext von Covid-19 beobachten. Appelle und Erklärungen ‚guter Hygiene‘ in Bezug auf Körper und Haushalt – richtiges Händewaschen, Desinfizieren von Händen und Flächen, das Einhalten einer physischen Distanz zwischen Personen, regelmäßiges Lüften und ein insgesamt besonnener Umgang mit der sozialen und häuslichen Umwelt – sind in fast allen Medienberichten zu dem neuartigen Virus zu finden (exemplarisch: Infektionsschutz 2020). Solche Anleitungen sollen aufklären, Einzelne und die Gemeinschaft schützen und Unsicherheiten abbauen – und erinnern zuweilen an Hygieneratgeber, die seit dem 19. Jahrhundert eine lange Tradition haben (Breuss 2006). Diskurse um die ‚richtige‘ Hygiene deuten zugleich auf soziale, politische und geografische Ungleichheitsverhältnisse hin. So wird unverkennbar, wer in welchen Regionen der Welt einen guten Zugang zu Gesundheitsleistungen hat, welches Land als fortschrittlich oder rückständig gilt. Spekulationen über Ursprünge und Formen der Ausbreitung des Virus können im Alltag umschlagen in rassistische Zuschreibungen und zu Diskriminierungen bis hin zu Gewalt führen.^2^

Hygiene und besonders Krankenhaushygiene gilt heute als Grundkonsens, der meist naturwissenschaftlich begründet wird. Wechselwirkungen zwischen medizinischem, sozialem und politischem Wissen sowie zwischen Hygienewissen und Herrschaftsmechanismen weisen jedoch eine lange historische Kontinuität auf. So war die hygienisch begründete Sauberkeit bereits zu Zeiten der Hygienebewegung vergeschlechtlicht und mit weiteren Differenzkategorien wie Klasse verwoben. War hygienische Aufklärung und die Einführung hygienischer Maßnahmen immer auch mit einer Disziplinierung von Arbeiter_innen und Distinktionspraktiken bürgerlicher Schichten verbunden, richteten sich Hygieneratgeber meist an Frauen als Zuständige für Haushalt und Familie (Breuss 2006). Die Verknüpfung von hygienischer Reinheit mit sozialen Zuschreibungen ging durch die bakteriologische Zuspitzung des Hygienediskurses im Alltag nicht gänzlich verloren (Sarasin 2001: 273; Vigarello 1992: 243). Wie wissensgeschichtliche Untersuchungen zum Aufkommen der Bakteriologie im 19. Jahrhundert deutlich machen, waren selbst die vermeintlich streng wissenschaftlich objektiven, im Labor gewonnenen Erkenntnisse der Bakteriologie zumindest anschlussfähig an rassistische Ideologien (Weindling 2007); das metaphorische Vokabular der Bakteriologie, so Phillip Sarasin, habe schon „von Anfang an ein rassistisches Imaginäres“ (Sarasin 2007: 453) in „den epistemischen Gegenstand ‚Bakterium‘“ (ebd.) eingeschrieben. Auch in zeitgenössischen Debatten um so genannte Krankenhausinfektionen, die während meiner Feldforschung besonders dominant waren, lassen sich wiederholt Imaginationen finden, die über im engeren Sinne medizinisches Wissen zu hygienischen Maßnahmen der Vorsorge und Behandlung hinausgehen und Dichotomien wie fremd und eigen, arm und reich, rückschrittlich und modern aufwerfen (von Bose 2017: 78–79).

Die heute weitgehende Verkürzung des Hygienebegriffs auf keimfreie Sauberkeit, die besonders in Krankenhäusern zu beobachten ist, lässt sich weder mit dem weiten Begriff von Hygiene der Hygienebewegung des 19. Jahrhunderts gleichsetzen noch mit Hygienediskursen zu Beginn der Bakteriologie (ausführlich von Bose 2017: 71–72). Das historisch gewachsene Wissen und die historisch kontinuierlichen Imaginationen um hygienische Sauberkeit bilden jedoch einen wichtigen Hintergrund, um aktuelle Aushandlungen der Krankenhaushygiene zu verstehen. Bevor diese näher beleuchtet werden, geht es zunächst um die Begriffe, mit denen sie analysiert werden sollen: Umweltfürsorge und Atmosphäre.

### Von der Umweltfürsorge zur Arbeit an der Atmosphäre

Joan Tronto and Berenice Fisher (1990) haben einen Carebegriff geprägt, der über das Verständnis von reiner Pflegetätigkeit hinausgeht und einen Umweltbegriff umfasst:

On the most general level, we suggest that caring be viewed as a species activity that includes everything that we do to maintain, continue, and repair our ‚world‘ so that we can live in it as well as possible. That world includes our bodies, our selves, and our environment, all of which we seek to interweave in a complex, life-sustaining web. (Tronto 1993: 103)

Diese Definition übersteigt die Vorstellung, die Care als in Beziehung zwischen Menschen stattfindende Fürsorge versteht, geschweige denn als institutionell eingebettete Krankenpflege in beruflich professionalisierten Settings. Maria Puig de la Bellacasa baut auf Trontos und Fishers Definition auf, um den Blick auf all das zu wenden, was bei einem intersubjektiven Careverständnis vernachlässigt wird: auf alltägliche Dinge und Tätigkeiten, die meist als unwichtig oder banal abgewertet werden, die aber zentral sind für das Zusammen- und Überleben (2011: 93). Sie etabliert damit nicht nur ein umfassenderes Verständnis von Fürsorge, das die Sorge um die Umwelt im Sinne von ökologischen Fragen einbezieht. Vielmehr geht es Puig de la Bellacasa auch darum, materielle Verhältnisse zu politisieren und womöglich umzukehren, unter denen Fürsorge stattfindet und Sorgearbeit verrichtet wird: „Caring, from this perspective, is a practice that most often involves asymmetry: some get paid (or not) for doing the care so that others can forget how much they need it“ (ebd.: 94). Ein solch weites Verständnis von Care, das eine Perspektive auf Fürsorge für die Umwelt einbezieht, bedeutet also auch Aspekten und Tätigkeiten Aufmerksamkeit zu widmen, die Fürsorge erst ermöglichen, transportieren, bedingen oder verhindern. Dies wird selbst dort virulent, wenn es um die sehr spezifische Form der Pflegearbeit an Menschen in institutionellen Kontexten geht.

Damit geraten im Krankenhaus nicht nur die in einem engeren Sinne unter Pflege subsumierten Arbeitsbereiche in den Blick, sondern auch Tätigkeiten der Hauswirtschaft und Reinigungsarbeiten. Dies ist nicht nur aus einer geschlechtertheoretischen Perspektive von Bedeutung, um die Relevanz dieser feminisierten Tätigkeiten für das Aufrechterhalten zentraler gesellschaftlicher Bereiche sichtbarzumachen, sondern ist darüber hinaus für die Erforschung von Wissenssystemen im Krankenhaus im Rahmen einer *hospital ethnography *(Geest & Finkler 2004; Strauss et al. 1963) zentral.

Unter der in Trontos Carebegriff einbezogenen Umwelt (*environment*) verstehe ich all das, was die menschlichen Beziehungen übersteigt, von diesen jedoch zugleich durchdrungen und konstituiert wird: der klinische Raum, der in Arbeitsprozessen erst hergestellt und affektiv-emotional aufgeladen wird, die Dinge, die diesen Raum ausmachen und bestimmte Handlungen erleichtern und andere verunmöglichen, sowie die Atmosphäre, die sich aus sozialen und nicht-menschlichen Akteur_innen zusammensetzt, nicht klar zuzuordnen ist, jedoch wahrnehmbar und verhandelbar erscheint und als medizinisch notwendig, hinderlich oder förderlich klassifiziert wird.

Der Begriff der Atmosphäre lässt sich als eine Zuspitzung des Umweltbegriffs verstehen. Mit ihm geraten besonders die affektiv-emotionalen Aspekte des *Cocooning* in den Blick – hier: der Fürsorge für die klinische Umwelt. Die Atmosphäre wurde in meiner Feldforschung immer wieder zum Thema, ob in Form eines Beklagens der bedrückenden Atmosphäre oder des schlechten Arbeitsklimas eines bestimmten Arbeitsbereichs oder eher implizit indem ein „Wohlfühlen“ in geputzten Räumen als Ziel der Reinigung gedeutet wurde (von Bose 2017: 133–134, 142–145). Atmosphären können als zugleich räumlich und subjektiv, affektiv und affizierend verstanden werden (Böhme 2007; Seyfert 2011) und sind von Hierarchieverhältnissen ebenso konstituiert wie von subjektiven Wahrnehmungen (von Bose 2019a: 316–317). Dafür ist ein Verständnis der Relationalität von Raum und Subjekt sowie die Beweglichkeit und Prozessualität von Raumkonstellationen zentral. Nach Michel de Certeau ist ein Raum als Ort zu verstehen, „mit dem man etwas macht“ (1988: 217). Räume sind weder einfach vorgegeben, noch immer gleich vorhanden, sondern ein „Geflecht von beweglichen Elementen“ (de Certeau 1988: 217): „So wird zum Beispiel die Straße, die der Urbanismus geometrisch festlegt, durch die Gehenden in einen Raum verwandelt“ (ebd.: 218). Ein Ort dagegen stelle eine „momentane Konstellation von festen Punkten“ (ebd.: 217–218) dar. Eine Station eines Krankenhauses besteht zwar aus bestimmten wiedererkennbaren Elementen – Patient_innenzimmer, Pflegestationen, Wartebereiche –, doch erst die alltäglichen Praktiken machen sie zu dem, was sie ist. So lassen sich die Räume nicht gänzlich auf die vorgesehene und offensichtlich erscheinende Funktion zurückführen, sondern erhalten im Alltag verschiedene Bedeutungen und Funktionen, werden unterschiedlich wahrgenommen, genutzt und angeeignet. Dies ist etwa zu beobachten, wenn eine Putzkammer temporär zu einem Aufenthaltsraum für eine Reinigungskraft wird. In der alltäglichen Bewegung immer wieder anderer Akteur_innen entstehen so auch immer wieder neue Räume, deren Analyse Aufschluss über die Logiken des Krankenhauses geben kann (von Bose 2017: 45–48). Betrachtet man die scheinbar banalen Arbeiten am Raum wie das Reinigen von Flächen und Gegenständen, kommen nicht nur so genannte patientennahe Tätigkeiten in den Blick, die für klinische Räume als wesentlich erachtet werden, sondern auch weniger beachtete Praktiken und ihre Akteur_innen, die an diesen Räumen und ihrer affektiven Wirkung – von Sterilität bis Komfort – mitarbeiten. Damit werden zudem größere, das Krankenhaus als Organisation bestimmende Prozesse erkennbar – von aktuellen Hygienediskursen über Ökonomisierungs- und Rationalisierungsprozesse bis hin zu den traditionell vergeschlechtlichten Arbeiten an der Umwelt der Patient_innen.

Der Begriff der Atmosphäre stellt besonders aufgrund seiner affektiven Dimension eine wichtige Zuspitzung des Umweltbegriffs dar. Denn damit wird deutlich, wie die Arbeit an der Umwelt spezifische Raumgefühle herstellt. So kann der Geruch einer Flächendesinfektion eine beruhigende Empfindung von Sauberkeit vermitteln (Washer & Joffe 2006), eine sichtbar schlecht oder nicht gereinigte Fläche dagegen Ängste oder Empörung bewirken. Die Sorge um eine wohltuende, gar heilende Atmosphäre verlangt wiederum nach ganz unterschiedlichen Tätigkeiten. Welche dabei in den Blick geraten (Architektur, Design) und welche meist vergessen werden (Reinigung, Pflege), ist ebenso feminisiert wie die Arbeit, die Fürsorge für Umwelten erfordert.

## Empirische Einblicke: Die feminisierte Arbeit an der Umwelt und Atmosphäre

In den folgenden empirischen Erkundungen steht die Frage im Vordergrund, welche Bedeutungen Sauberkeit und Hygiene bei der Arbeit an der Atmosphäre der Räume jeweils erhalten und wie die Zuständigkeiten dafür ausgehandelt werden. In den Analysen geht es sowohl um die soziale Herstellung und Wirkung benennbarer Orte – Bett, Zimmer, Station –, als auch um das Nachvollziehen sozialer „(An)Ordnungen“ (Löw 2001), die Raumstrukturen stets neu hervorbringen und an denen alltäglich gearbeitet werden muss. Am Bett und im Zimmer sind vor allem Reinigungs- und Pflegekräfte zugegen, beim Blick auf die Station als gesamten Raum geraten weitere Akteur_innen wie Ärzt_innen und Architekt_innen ins Blickfeld.^3^

### Das Krankenbett. Von Intimität und Kontrolle, textilen Bakterienfängern und dem Aushandeln von Arbeitsbereichen

Mit Roland Barthes lässt sich das Krankenbett als Proxemie begreifen, als ein „Mikroraum“ (2007: 185), der die unmittelbare Umgebung des Subjekts bildet. Barthes bezeichnet das Krankenbett sogar als die „stärkste, am intensivsten erlebte, oftmals bestorganisierte Proxemie“ (ebd.: 187). Um 1900 wurde das Krankenbett im Zuge der Verordnung von Bettruhe selbst für psychisch Erkrankte zu einem Ort langen Aufenthalts. Wie Monika Ankele anhand von Krankenakten und Selbstzeugnissen psychiatrischer Patientinnen im ausgehenden 19. Jahrhundert zeigen kann, wurde das Bett hier zur Zuflucht, zum Rückzugsort und Raum unterschiedlichster Aneignungspraktiken am „Ort des Anderen“ (2010), der totalen Institution. Karin Harrasser wiederum analysiert das Krankenbett als Raum der Patient_inwerdung, als „Artikulations- und Blickmaschine, einen geschichteten Raum, ein Dispositiv im Foucaultschen Sinne“ (2012: 234). Er diene zum einen der Schonung und des Schutzes der Patient_innen, zum anderen aber auch der Disziplinierung, sei einerseits „Refugium der Intimität“ (ebd.: 236), andererseits „Schauraum für Ärzte, Krankenhauspersonal, andere Kranke, Familie und Freunde, aber auch als Ort der Datensammlung, des (Nicht)Wissens, der unsicheren Entscheidungen“ (ebd.).

Stellt das Bett im Krankenhaus also den intimsten Raum für Patient_innen dar, wird er doch zugleich durch unterschiedlichste Akteur_innen der Krankenhausarbeit konstituiert. Im empirischen Material meiner Studie gerät das Bett vor allem aus Perspektive derjenigen in den Blick, die es nicht bewohnen (müssen), sondern an es herantreten, an ihm tätig werden, um es herum ordnen und reinigen. Seine Bedeutung erscheint dabei umgekehrt: Nicht die Verletzbarkeit des liegenden Körpers der zur Patient_in gewordenen Person gerät hier in den Fokus, sondern die Arbeit am Bett und die zuweilen verletzenden, prekären Bedingungen, unter denen sie verrichtet wird.

Aus Perspektive von Reinigungskräften kann das Krankenbett im Certeau’schen Sinne zu einem Raum der Überwachung werden, in Harrassers Verständnis zum Schauplatz der Disziplinierung. Hier betrifft die Überwachung jedoch nicht die Patient_innen selbst, sondern geschieht aus Sicht der Reinigungskräfte seitens der Patient_innen: „Also bei den Patientenzimmern ist es allerdings so, die warten irgendwie auf dich. Wenn die merken, du bist einen Tag da nicht drin gewesen, dann meckern die schon gleich.“ (Interview Frau A.) Patient_innen kontrollierten vom Bett aus die Arbeit der Reinigungskräfte: „[…] da liegen Patienten, die beobachten, was die Reinigungsfrau macht. Weil, die liegen im Bett, die haben Langeweile, die gucken ganz genau, wie man sich bewegt, was man macht, welchen Lappen man in der Hand hat, welches Reinigungszeug.“ (Interview Frau B.). Zusätzlich werde von Patient_innen besonders viel Schmutz produziert, so dass sich eine höhere Dringlichkeit für die Reinigung ihrer Zimmer ergebe als in anderen Abteilungen des Krankenhauses: „Es staubt ja auch viel, die bewegen sich da viel und so weiter, du hast diesen engen Zeitplan und du musst da jeden Tag rein und hast so viel, also musst du wirklich rennen, wenn du alles überall schaffen willst.“ (Interview Frau A.) Die Patient_innen treten hier aus ihrer Rolle als passive Objekte des medizinischen Blickes heraus, werden selbst zu Verursachenden von Schmutz und Kontrollinstanzen der Reinigungsarbeit. Dies wird vor dem Hintergrund umso relevanter, als die Reinigung in dem untersuchten Krankenhaus an ein Tochterunternehmen ausgelagert ist, das enge Raum- und Zeitkontingente vorgibt, die einen hohen Zeitdruck produzieren. So müssen beim Reinigen Prioritäten gesetzt und es muss hier und da „getrickst“ werden, um das Pensum überhaupt annähernd zu bewältigen. Der Blick der Patient_in wird in diesem System zu einem der Kund_in, unter dem die Arbeit der Reinigungskraft als Service erscheint, der vermisst, eingefordert und bemängelt werden kann – was unter Umständen die Arbeitsstelle der ohnehin meist befristet angestellten Reinigungskraft gefährdet.

Während Reinigungskräfte vorwiegend die Umgebung von Krankenbetten bearbeiten, finden Kernaufgaben von Pflegekräften am Krankenbett selbst statt – das Gespräch mit der Patientin, die konkreten pflegerischen Handgriffe wie das Versorgen von Operationswunden, Anschließen von Infusionen, Mobilisieren bis hin zur Körperpflege von Patient_innen. Das Bett und seine Materialien werden dabei alltäglich zu einem Gegenstand von Aushandlungen der stationären Arbeitsteilung, in die auch Reinigungskräfte involviert sind. Eine Reinigungskraft schildert mir, wie Pflegekräfte einer Station aus ihrer Sicht schrittweise Arbeit an sie abgegeben haben:

Und dann fingen sie an, die Betten nicht mehr abzuziehen. Normalerweise ziehen die ja selber die Betten ab und stopfen das schmutzige Bettzeug in diesen Wäschesack rein. Noch nicht einmal die Betten haben sie mehr abgezogen, haben alles für mich liegen gelassen. Also mir immer mehr zugeschoben. (Interview Frau A.)

Diese Aufgabe implizit übertragen zu bekommen, bedeutet für die Reinigungskraft nicht nur eine ärgerliche Mehrarbeit, sondern kann darüber entscheiden, ob sie ihr Arbeitspensum bewältigt oder an anderer Stelle Abstriche oder unbezahlte Überstunden machen muss. Statt dies hinzunehmen, wehrt sie sich mit dem Verweis auf die hygienischen Gefahren, die beim Abziehen eines Bettes entstehen können:

Das habe ich aber nicht gemacht, habe gleich gesagt, tut mir leid, das ist eure Aufgabe […]. Denn ob die die Bettwäsche anfassen oder ich die Bettwäsche anfasse, irgendwie, und Bettabziehen war noch nie meine Aufgabe. Und die sind ja vorher persönlich mit dem Patienten in Tuchfühlung gekommen, ich ja noch nicht einmal, also können sie auch die Bettwäsche anfassen. (Ebd.)

Die Charakterisierung der Bettwäsche als potentielle Gefahrenquelle reiht sich in Einschätzungen medizinischen Personals ein, die Textilien nach ihrer hygienischen Bedeutung klassifizieren. Gelten Kitteltaschen medizinischen Personals als besonders „verkeimt“, bezeichnete eine Pflegekraft mir gegenüber das Krankenbett als „Bakterienfänger“. Meine Gesprächspartnerin Frau A. beruft sich also auf ein geteiltes stationäres Hygienewissen, um eine Aufgabe zurückzuweisen, die nicht offiziell in ihren Bereich fällt. Dass Hygienewissen zu einem Argumentationsinstrument wird, um Arbeitsbedingungen zu politisieren oder im Alltag auf der vereinbarten Arbeitsteilung zu bestehen, konnte ich während meiner Forschung vielfach beobachten (von Bose 2017: 179–272). Dass es für eigene Zwecke genutzt wird, schmälert jedoch nicht die Relevanz des Arguments selbst. Das Wissen, auf das Frau A. sich hier bezieht, ist ihr Erfahrungswissen aus dem Krankenhaus, das Infektionsgefahren an die Nähe zu kranken Körpern koppelt – und damit nicht nur einem krankenhaushygienischen Wissen, sondern auch affektiven Wahrnehmungsmustern folgt. Je intimer ein Gegenstand mit einer als solcher eingestuften Gefahrenquelle in Berührung gekommen ist, desto eher entstehen angesichts der Berührung dieses Gegenstands Gefühle von Angst oder Ekel (Ahmed 2004: 83). An anderer Stelle im Interview äußert Frau A. ihre Bewunderung für den Pflegeberuf, die Verantwortung und die Gefährdungen, die er mit sich bringt; sie ist jedoch nicht bereit, diese mitzutragen. Um sich zu schützen, lehnt sie die Aufgabe ab. Der Begriff, den sie dafür verwendet, könnte in seiner textilen Assoziation treffender nicht sein: Die Pflegekräfte seien mit den Bewohner_innen des Bettes und seiner Wäsche bereits auf „Tuchfühlung“ gewesen. Die Fürsorge für die unmittelbare Umwelt der Patient_innen, für die Tuchfühlung, weist Frau A. damit von sich.

Das Krankenbett zeichnet sich also auf unterschiedliche Weise durch Intimität aus. Wird es für Patient_innen zu einem Raum, der zwischen Intimität und Öffentlichkeit changiert (Harrasser 2012), geht von ihm für Reinigungskräfte sowohl eine Gefahr der Kontrolle ihrer Arbeit als auch der Ansteckung aus. Die „Tuchfühlung“, die für Patient_innen in Form der Bettdecke schützend wirken kann, schlägt in der Arbeit mit potentiell infektiösen Textilien in eine unangenehme Nähe um. Das Bett wird dabei auch zum Ort der Aushandlung über die Zuständigkeit für einzelne Tätigkeiten.

An diesen Aushandlungsprozessen am Krankenbett lassen sich Hinweise auf die Prekarität dieser feminisierten Fürsorgearbeit ablesen: Während sich Patient_innen zu Recht ein hygienisches Umfeld wünschen, ist die Arbeit daran von Ökonomisierungsprozessen im Krankenhausmanagement ebenso geprägt wie von konfliktreichen Aushandlungen um die Arbeitsteilung. Dass diese Arbeit feminisiert und ethnisiert ist, wird nur implizit und durch Kontextwissen deutlich: Indem „alle“ sich beschweren und über Techniken und Materialien des Reinigens urteilen können („die gucken ganz genau […] welchen Lappen man in der Hand hat, welches Reinigungszeug“), wird die Krankenhausreinigung als hausarbeitsnahe Tätigkeit markiert, die traditionell feminisiert ist (Thiessen 2004; Anderson 2000), und ihr wird ein Expert_innenwissen aberkannt; Reinigungskräfte haben zudem als meist migrantisch markierte Frauen und aufgrund der Politik des Outsourcing einen so niedrigen Status in der Klinikhierarchie, dass eine Zurückweisung zusätzlicher oder gefährdender Arbeit, wie die zitierte Reinigungskraft sie beschreibt, erheblich erschwert wird.

### Das Krankenzimmer. Von feminisierter Reinlichkeit und Reinigung als Umweltfürsorge

Das Krankenzimmer stellt einen weniger intimen, körpernahen Raum dar als das Bett. Die Arbeit an der Umwelt der Krankenbetten kommt stärker in den Blick. Doch auch hier changiert die Raumwahrnehmung zwischen öffentlich und privat, zwischen dem persönlichem Nahraum der Patient_innen und der institutionalisierten Raumpflege durch Krankenhauspersonal. Im Folgenden geht es weiter darum, wie die Aushandlungen um die Zuständigkeit für diese Umweltfürsorge auf unterschiedliche Weise (und historisch kontinuierlich) vergeschlechtlichend wirken.

„Eigentlich würden sie es gerne sehen, wenn wir das täten. Aber so weit sind wir dann doch nicht, dass wir auch noch die Blumenpflege übernehmen“ (Interview Frau H.), so erklärt mir eine Pflegerin eine typische Kontroverse zwischen Pflegekräften und Patient_innen um das Versorgen mitgebrachter Blumensträuße. Frau H. ordnet diese Forderung in eine allgemeine Anspruchshaltung heutiger Patient_innen ein, deren Anforderungen an die Pflege Frau H. entschieden zurückweist. In ihren Schilderungen solcher Situationen klingt die Kritik an einer Konsum- und Dienstleistungslogik an, die aus ihrer Perspektive den Krankenhausalltag immer mehr bestimmt:

Im Allgemeinen sind die Patienten ja sehr verändert in der Persönlichkeit. […] Also früher, […] da haben die ihren Nachttisch als ihr unmittelbares, ja wie zu Hause der Nachttisch, da haben die sich verantwortlich für gefühlt und haben den gereinigt. Sie selber, ne. Ihre Blümchen gemacht. […] Manche halten den ganz sauber. Das ist auch bis heute, aber das war früher generell, dass die Patienten sich ihr Umfeld, das haben sie sauber und ordentlich gehabt […]. (Interview Frau H.)

Sich um den eigenen Nachttisch und die eigenen Blumen zu kümmern, wurde früher von allen Patient_innen selbst übernommen, so ihre Beobachtung. Frau H. beschreibt dieses Früher als eine Zeit, zu der alle Patient_innen noch sorgsam, ordentlich und reinlich mit ihrer Umgebung umgingen. Sie nahmen ihre persönlichen Pflichten im Krankenhaus genauso wahr wie zu Hause. In dieser Beschreibung wird die veränderte Haltung von Patient_innen als „Persönlichkeit“ individualisiert. Sie ist jedoch in den größeren Kontext der Ökonomisierung des Krankenhaussektors einzuordnen (Mohan 2019), durch die nicht-medizinische Leistungen im Krankenhaus auch in den Augen von Patient_innen als Service wahrgenommen werden. Wie anhand der Komfortstation später noch deutlich wird, müssen sich Krankenhäuser in Zeiten des Wettbewerbs immer mehr an Hotelstandards orientieren (Behar et al. 2018: 207), was wiederum auf Patient_innen und ihre Ansprüche zurückwirken kann.^4^ Indem Frau H. jedoch eben nicht einen Kontext der Dienstleistung, sondern den Privathaushalt („zu Hause“) als Referenzkontext einführt, erscheint die Unordnung der Nachttische als persönliches Versäumnis der Patient_innen, die ihrer Zuständigkeit nicht nachkommen. In ihrer Deutung wird nicht nur das Bett, sondern in seiner Verlängerung auch der Nachttisch zu einem privaten Raum, für dessen Sauberkeit, Ordnung und Blumenschmuck allein die ‚Bewohner_innen‘ zuständig sind.

Das Zurückweisen der Zuständigkeit für die Blumenpflege im Krankenzimmer seitens der Pflegekraft lässt sich als Zurückweisen eines *Cocoonings* interpretieren, das sie im Aufgabenbereich der Patient_innen verortet und von ihnen einfordert. Dass diese Aufgabe dabei eine feminisierte bleibt, ist vor dem Hintergrund zu verstehen, dass sich Frau H.s Beobachtungen auf eine gynäkologische Station beziehen, auf der sie seit mehreren Jahrzehnten arbeitet. Dies ist in ihrer Sprache nicht zu erkennen („Patienten“), stellt für die Deutung aber ein wichtiges Detail dar: Würde sie auch männlichen Patienten mangelnde Reinlichkeit und Unordnung vorwerfen? Die Verknüpfung von Reinlichkeit mit Weiblichkeit ist jahrhundertealt (Kelley 2010) und bezieht sich sowohl auf eine weibliche Zuständigkeit und Kompetenz für Sauberkeit sowie für das Private, als auch auf das Aufrechterhalten von Respektabilität durch (weiße) Frauen (Schmidt 2017: 69).^5^ Victoria Kelley stellt etwa für das viktorianische England fest: „Cleanliness has often been associated with feminine virtue of the domestic sort, a visible symbol of both competence and propriety“ (2010: 14).^6^

Patient_innen, die ihr persönliches Umfeld nicht sauber und ordentlich halten, fallen in Frau H.s Augen offensichtlich aus dieser (weiblich konnotierten) Respektabilität heraus. Durch den Bezug auf den Privathaushalt wird diese Sorge für das unmittelbare Umfeld zu einer Privatangelegenheit, für die sie als Angestellte des Klinikums die Zuständigkeit legitim zurückweisen kann. Die traditionell vergeschlechtlichte Grenzziehung zwischen privat und öffentlich, gemäß derer Frauen der privaten Sphäre zugeordnet werden, dient ihr hier implizit dafür, sich gegen die Zumutung zu wehren, sich angesichts der Fülle ihrer Aufgaben und der Hektik des Alltags auch noch Dingen widmen zu müssen, die wie typische Hausarbeiten erscheinen. Die Ausführung solcher haushaltsnahen Tätigkeiten ist nämlich mit einer Abwertung verbunden, mit der der Pflegeberuf nach wie vor konfrontiert ist: „Like many occupations, nursing work, women’s work and dirty work are inextricably linked due to an association with the private realm“ (Bolton 2005: 170).

Zugleich wird hier auch ein Zurückweisen der Zuschreibung deutlich, für all die Aufgaben zuständig zu sein, die nicht klar zugewiesen sind: „Also wir füllen immer diese ganzen Lücken. Jeder weiß immer ganz genau, was er darf. Nur die Krankenschwester, die weiß nicht genau, was sie darf. Die macht alles das, was übrigbleibt.“ (Interview Frau G.; von Bose 2017: 201–202) Diese schwammige Zuständigkeit für „alles das, was übrigbleibt“ knüpft an ein traditionelles Verständnis von Pflege als umfassende Sorgetätigkeit an. In der Bezeichnung „Krankenschwester“ wird die Geschichte der Krankenpflege als christlich geprägtes Berufsbild sichtbar:

Das Arbeitsethos des aufopferungsvollen „Liebesdienstes“ basierte auf dem christlichen Gebot der Barmherzigkeit. Indem sich die Schwestern den kranken und bedürftigen Menschen widmeten, zeugten sie von der Liebe Gottes und nahmen am Aufbau seines Reiches teil. So gesehen verstand sich die Krankenpflege nicht in erster Linie als medizinischer Assistenzberuf, sondern in hohem Maße als religiöser Auftrag. (Kreutzer 2005: 17)

„Aufopferungsvoll“ war der Beruf noch zu Beginn der 1950er Jahre nicht nur deshalb, weil er als unvereinbar mit einem eigenständigen Leben – Ehe, Familie, unabhängiger Wohnort außerhalb von Wohnheimen – jenseits der Pflegetätigkeit galt, sondern auch, weil das Aufgabengebiet bislang kaum ausdifferenziert war.^7^ Eine Aufteilung in hausarbeits- und medizinnahe Tätigkeiten mit entsprechendem Status gehörte zu den wesentlichen Elementen der immer weiter fortschreitenden Professionalisierung (ebd.: 28). Die Modernisierung und langsame Abwendung von dem Ideal einer Krankenpflege als (allumfassender, lebensbestimmender) Liebesdienst wurde nicht zuletzt durch einen starken Personalmangel in der Pflege angestoßen und ging sowohl mit einer Öffnung des Berufsfelds für Männer als auch mit der Anwerbung ausländischer Fachkräfte einher (ebd.: 24–33). Das Ringen darum, den Pflegeberuf aus seinen feminisierten Konnotationen herauszulösen und damit zugleich aufzuwerten, hat also eine lange Geschichte.

Die Anforderung, Patient_innen nicht nur physisch zu versorgen, sondern ihnen mit „tender loving care“ (Strauss et al. 1985: 129) rundum beizustehen, scheint jedoch im heutigen Pflegealltag nach wie vor präsent zu sein. Der in meiner Studie häufig geäußerte Wunsch von Pflegekräften, mit ihrem medizinischen und professionellen Praxiswissen ernster genommen zu werden, wirkt vor dem historischen Hintergrund wie eine Abgrenzung von eben dem Bild der feminisierten Schwester, deren Arbeit als typisch weiblich naturalisiert und damit entwertet wurde (McDowell 2009: 165). Die Tragweite alltäglicher Aushandlungen um Zuständigkeiten bis hin zu der Frage, wer die Blumen von Patient_innen versorgt, werden erst vor diesem historischen Hintergrund gänzlich nachvollziehbar. Durch die Abgrenzung zwischen haushaltsnahen und medizinisch-pflegerischen Tätigkeiten wird diese vergeschlechtlichte Differenz und Wertzuschreibung zwischen privater und öffentlicher Sphäre jedoch auch immer wieder hergestellt (auch von Bose 2019a).

Dass sich diese Aspekte (haushaltsnah und medizinisch) auch in ihren Effekten kaum voneinander trennen lassen, wird besonders anhand der hygienischen Relevanz der Reinigung deutlich. Eine Pflegerin erklärt mir im Interview, wie die Flächenreinigung direkt mit der hygienischen Qualität ihrer pflegerischen Arbeit zusammenhänge: „Ja, natürlich hast du Einfluss auf die sterile Arbeit, aber eigentlich kannst du nie hundertprozentig sauber arbeiten, weil unter den Betten liegen die Flocken, auf dem Nachttisch liegen die Flocken und das stört mich schon.“ (Interview Frau H., auch von Bose 2017: 119) Sie spielt damit auf die Verschlechterung durch Sparmaßnahmen im Bereich der Reinigung an, die von allen angesprochen wurden, mit denen ich während meiner Forschung sprach, von Ärzt_innen über Pflege- bis zu Reinigungskräften selbst. Die Staubflocken in Frau H.s Schilderung markieren eine Begrenzung der pflegerischen Einflussnahme auf das sterile oder zumindest saubere Arbeiten und die Reinigung erhält eine direkte Relevanz für die hygienisch korrekte Versorgung der Patient_innen. Zugleich steht der Staub auch sinnbildlich für eine (strukturell begründete) Vernachlässigung der Räume, die Frau H. auch emotional zu berühren, zu frustrieren scheint.

Solchen Schilderungen widersprechen die Ansprüche von Reinigungskräften selbst, mit denen ich sprach. Die Reinigungskraft Frau A. sieht den Sinn ihrer Arbeit darin, dass sich die Nutzer_innen der von ihr geputzten Räume „wohlfühlen“: „Wenn es nicht sauber ist, ist es ungemütlich, nicht schön. Dass es sauber ist, dass sich die Leute wohl fühlen können, oder für die Besucher oder für die Patienten, das ist mir schon wichtig“ (Interview Frau A.). Sie markiert ihre Arbeit damit als Fürsorgearbeit – ganz im Sinne eines umfassenden Carebegriffs (Puig de la Bellacasa 2011). Statt lediglich störenden Schmutz zu entfernen, erschafft sie eine Atmosphäre, die für andere wohltuend ist (weiterführend von Bose 2019a). Damit deutet sich an, was im Folgenden ausgeführt wird: Die Reinigungsarbeit trägt wesentlich zu den entstehenden Raumgefühlen bei, es geht nicht nur um das Herstellen einer sauberen, keimfreien Umgebung der Patient_innenversorgung, sondern zugleich um das Hervorbringen eines angenehmen Gesamteindrucks der Station – dessen Akteur_innen jedoch meist unbemerkt bleiben.

### Die Krankenstation. Von Ästhetik, Hygiene und Differenzsetzungen bei der Arbeit an der Atmosphäre

Während es bei der Arbeit an der direkten Umwelt des semi-privaten Krankenbetts im Krankenzimmer vorwiegend darum ging, die Zuständigkeit für und vergeschlechtlichte Bewertung von Aufgaben der Umweltfürsorge auszuhandeln (wer wo und auf welche Weise reinigt, ordnet, verschönert), geht es auf der Ebene der Station in einem weiterführenden Sinne um die Arbeit an der Stationsatmosphäre. Während klinische Räume wie der Operationssaal eher Ärzt_innen zugeordnet sind, ist die Krankenstation der Hoheitsbereich der Pflegekräfte. Dies realisiert sich, so die These dieses Abschnitts, nicht zuletzt in der Arbeit an der Atmosphäre der Station. Wie deutlich werden wird, zielt die Atmosphäre auf ein Zugleich von Ästhetik und Hygiene und damit verbunden: Prestige.

Einige Pflegekräfte drückten mir gegenüber ihre Bedenken über den Eindruck aus, den Patient_innen und Besucher_innen schon beim Betreten des Eingangsbereichs von der Station erhielten. Die Station wirke durch die im Eingang abgestellten Servicewagen der Speiseversorgung sowie Wasserkästen und Teewagen bereits bei Betreten der Station unordentlich und unsauber. In dieser Sorge um den negativen Gesamteindruck, den andere von der Station erhalten könnten, drückt sich zugleich ihr Gefühl der Zugehörigkeit zur Station aus. Häufig fallen Begriffe wie „bei uns“, die den Stationsraum als ihren eigenen markieren, als ihren Wirkungsort herstellen (von Bose 2017: 116–122).

In dieser Funktion agieren sie auch als Vermittler_innen zwischen Patient_innen, die sich über den Zustand der Räume beschweren, und den zuständigen Instanzen wie Reinigungskräften und dem Krankenhausmanagement. Dabei reflektieren sie immer wieder die strukturellen Bedingungen insbesondere der Reinigung im zeitlichen Verlauf, wie sie bereits im Krankenzimmer Thema waren. Im Interview erklärt mir der Pfleger Herr I. seine Perspektive auf den Unterschied zwischen der Arbeit der Reinigung, wie sie heute organisiert ist, und den Zuständen vor einigen Jahren, als diese noch nicht an das Tochterunternehmen des Krankenhauses ausgelagert worden war:

Also ganz früher, das war ja keine Fremdfirma, die früher geputzt hat, das waren Stammkräfte auf der Station. Die eine war vormittags da, die andere nachmittags. Und, ich muss dazu sagen, es war damals noch deutsches Personal. Die haben sich verbunden gefühlt mit der Station, die haben geguckt: Meine Station ist sauber. Die haben aufgeräumt, abgewischt und die Patienten noch mitbetreut und haben da immer gefragt, ob sie was zu trinken brauchen. Und das kommt ja bei diesem Personal, die jetzt saubermachen, nicht mehr in Frage, weil die kommen ja nur zum Saubermachen und deswegen blieb ja alles an uns hängen. (Interview Herr I.)^8^

Auffallend ist hier, dass er in seiner Darstellung einer klaren Verschlechterung im Bereich der Reinigung durch die Sparpolitik des Outsourcings zwar auf strukturelle Aspekte eingeht, vor allem aber auf einer sozialen Ebene argumentiert. Die „Stammkräfte“ waren früher fest für eine Station zuständig und insgesamt in Vollzeit anwesend, haben nicht wie heute häufig ihren Einsatzort gewechselt oder waren als Einzelperson für mehrere Stationen zuständig. Statt aber weiter die Arbeitsbedingungen in der Reinigung zu kritisieren, beleuchtet er hier mehr die individuellen, für ihn unmittelbar beobachtbaren Praktiken des Reinigungspersonals. So betont er die Bereitschaft der früheren „Stammkräfte“, über den eigenen Aufgabenbereich hinaus an Stationsaufgaben mitzuarbeiten, indem sie sich auch an der Arbeit der Pflege beteiligten und eine klare Entlastung für ihn und seine Kolleg_innen darstellten. Sie räumten auf und fragten Patient_innen nach ihren Wünschen. Diese Fürsorge galt in Herrn I.s Augen der ganzen Station: „Die haben sich verbunden gefühlt mit der Station, die haben geguckt: Meine Station ist sauber.“

In Herrn I.s Gegenüberstellung eines Früher und Heute der Reinigung mischt sich ein rassistischer Unterton, der die Verbundenheit der Reinigungskräfte zur Krankenstation mit einer nationalen Zugehörigkeit verbindet. Bereits der Begriff „Fremdfirma“ konstituiert eine Differenz, in der das Krankenhaus und sein „Stamm“-Personal als das Eigene markiert wird, hingegen das Tochterunternehmen der Klinik als das „Fremde“ erscheint. Indem der Pfleger anschließend betont, dass es sich damals noch um deutsches Personal handelte, verbindet er diese Differenz zwischen eigen und fremd mit einer nationalen Zuschreibung. Hier wird zwar nicht explizit und unmissverständlich ausgesprochen, dass deutsche Reinigungskräfte im Gegensatz zu als nicht-deutsch wahrgenommenem Personal gründlicher seien. Ebenso wenig wird eine direkte Verbindung zwischen als fremd wahrgenommenen Personen und Unreinheit oder Schmutz gezogen. Die diskursive Nähe jedoch, die zwischen dem Begriff „Fremdfirma“ und der Schilderung einer ungenügenden Reinigung besteht sowie zwischen den damals noch deutschen Reinigungskräften und deren besserer Sorge für die gesamte Station, nimmt Verknüpfungen von dem (national konstruierten) Eigenen mit Sauberkeit sowie dem (national zugeschriebenen) Anderen mit Schmutz, Unreinheit und potentiellen Infektionsgefahren auf. Diese Verknüpfungen weisen für das Hygienethema wie eingangs geschildert eine lange historische Tradition auf (Sarasin et al. 2007) und werden auch im heutigen Klinikalltag immer wieder reproduziert (von Bose 2017: 262–272).

Die tägliche Arbeit an der Verbesserung des Gesamteindrucks der Station obliegt nun nicht (mehr) nur den Reinigungskräften, sondern umfasst auch für die Pflege ganz unterschiedliche Tätigkeiten. Ein so genanntes Putzbuch regelt auf der Station meiner Feldforschung die Verteilung von Reinigungs- und Aufräumarbeiten, die nicht von der Reinigungs- oder Servicekräften übernommen werden, weil sie entweder nicht der Institution zuzurechnen sind oder weil sie aus formalen Aufgabenverteilungen herausfallen – beispielsweise das Spülen der eigenen Kaffeetassen, das Auswischen des gemeinschaftlich genutzten Kühlschranks oder Aufräumen des Wartezimmers. Eine der Pflegerinnen hängt zur Weihnachtszeit selbst gebastelte Sterne auf, um die Station etwas freundlicher wirken zu lassen, obwohl das gegen die Hygienevorgaben verstößt. Geht es um die Verbesserung der negativ konnotierten ‚Krankenhausatmosphäre‘, so werden zuweilen auch Hygieneregeln umgangen, um der gefühlten Sterilität entgegenzuwirken.

Solche Praktiken verweisen auf die Logiken des *Cocooning*: Die Fürsorge für einen freundlich wirkenden Raum ist eine feminisierte, unsichtbare und liegt teilweise sogar quer zum medizinisch anerkannten Hygienewissen. Zählen all solche Aufgaben zu feminisierten haushaltsnahen Tätigkeiten, nehmen die Pflegekräfte, die ich bei ihrer Arbeit begleitete, dabei jedoch keine Geschlechterzuordnung vor. Im Gegenteil, der einzige männliche Pfleger einer Station betonte, dass er fast reinlicher und penibler bei seinen Putzaufgaben sei als die „Schwestern“. Allerdings wurden Grenzen zu „den Ärzten“ gezogen, die jedoch auf der gynäkologischen Station, auf der ich den Großteil meiner Feldforschung verbrachte, zumindest auf Ebene der Stationsärzt_innen vorwiegend Frauen waren. Ob über die mangelnde Sauberkeit des Kühlschranks im Ärztezimmer gesprochen wurde („also da läuft dir manchmal was entgegen“) oder scherzhafte Aussagen gemacht wurden wie „Ärzte waren noch nie für Aufräumen“ – immer wieder wurde implizit oder explizit das mangelnde Bewusstsein für Sauberkeit und Ordnung seitens der Ärzt_innen ausgedrückt. In diesen Kommentaren deutet sich die hierarchisierte Arbeitsteilung im Krankenhaus an, die damit nicht lediglich ausgedrückt, sondern immer wieder hergestellt wird. Der sorgende Blick für die Sauberkeit und Ordnung der Station, den die Pflege zu haben scheint, Ärzt_innen dagegen weniger, und der auch mehr oder weniger formal der Pflege zugeordnet ist, deutet zugleich auf eine Bewertungshierarchie hin: zwischen jenen Arbeitsbereichen, die sich auf die medizinische Behandlung konzentrieren können, und denen, die auch für die Umwelt der medizinischen Behandlung sorgen müssen.^9^ Der nach wie vor feminisierte Beruf der Krankenpflege ist in der Klinikhierarchie zwar den Ärzt_innen nebengeordnet, steht im Status jedoch nach wie vor unter ihnen, was sich in den ganz alltäglichen Aufgabenverteilungen konstituiert und reproduziert. Wenn ein Oberarzt in der Pause der Pflegekräfte in deren Aufenthaltszimmer stürmt und die Runde auf unwirsche Weise ermahnt, doch bitte das Wartezimmer aufzuräumen, dann zeigt sich, dass es sich hier um eine klare Hierarchie handelt.^10^ Die Vergeschlechtlichung der Aufgabenverteilung muss sich dabei nicht unbedingt in der Geschlechterverteilung der konkreten Akteur_innen niederschlagen. Sie ist der Arbeitsteilung jedoch eingeschrieben, die Umweltfürsorge an Weiblichkeit koppelt. Dass ein männlicher Pfleger eigens betonen muss, dass er sogar reinlicher sei als seine weiblichen Kolleginnen, deutet ebenso auf die nach wie vor bestehende feminisierte Konnotation von Reinlichkeit hin (von Bose 2017: 120–122).

Allerdings geht es bei der täglichen Sorge um die Station als Umwelt der medizinischen Versorgung von Patient_innen keineswegs lediglich um eine feminisierte Form der Reinlichkeit oder Raumpflege wie in einem häuslichen, privaten Kontext. Denn die meisten Arbeiten, die sich auf die eine oder andere Art dem Stationsraum widmen, haben implizit oder explizit mit der Krankenhaushygiene zu tun. So hatten sich die Pflegekräfte der beforschten Station über einen längeren Zeitraum hinweg um eine Grundreinigung der Böden der Station bemüht, die eine Desinfektion miteinschloss und – als sie schließlich genehmigt und durchgeführt worden war – den Boden sichtlich aufhellte. Die Reinigung umfasste also beides: eine Verbesserung der Ästhetik und Hygiene. Die Relevanz dieser Gleichzeitigkeit liegt auch an der Wahrnehmung von Patient_innen, die aus Erfahrung der Pflege kaum zwischen optischer Sauberkeit und hygienischer Reinheit unterscheiden können: „Wenn der Boden optisch hell ist, dann empfinden die das als hygienisch rein.“ (Interview Frau G.) Ihr medizinisches Wissen sagt ihr, dass sich auf einer gewischten Fläche dennoch unsichtbare Keime befinden können; optisch saubere Flächen seien aber für das Sauberkeitsempfinden von Patient_innen schon ein Gewinn. Sichtbarer Schmutz dagegen verleitet zu Hochrechnungen: wenn da schon Staubflocken liegen, kann es ja nicht sauber sein und damit schon gar nicht hygienisch rein. Es ist also stets ein Zugleich beider Elemente – sichtbare Sauberkeit und unsichtbare Keimfreiheit.

Der Zusammenhang zwischen einem ästhetischen Gesamteindruck und dem Vertrauen in die medizinische Versorgung, der sich in der Sorge der Pflegekräfte um den Ruf ihrer Station ausdrückt, wird von Studien aus der Verhaltenspsychologie bestätigt:

If patients notice that health care providers (or someone connected to them) put time, thought, and care into the hospital environment, such actions may be interpreted to mean these providers care for patients’ well-being and comfort. As a result, patients may expect those providers will put the same quality into the given ‚technical‘ care. (Campos Andrade et al. 2016: 303)

Insofern als Schmutz in all seinen möglichen Formen zu einer (gefühlten) Bedrohung durch unsichtbare Krankheitserreger und Infektionsgefahren werden kann, umfasst das Engagement für einen angenehmen Eindruck stets auch den Einsatz für das Vertrauen in eine gesundheitsfördernde Station (auch von Bose 2017: 122). Dass diese Gleichzeitigkeit jedoch nicht dazu führt, dass die Bewertungshierarchie zwischen Aufgabenbereichen zugunsten solcher Fürsorgetätigkeiten nivelliert würde, ist eine der Paradoxien der vergeschlechtlichten Arbeitsteilung (weiterführend von Bose 2019a).

Ein Blick in eine so genannte Komfortstation eines anderen Krankenhauses verdeutlicht abschließend den Zusammenhang zwischen Arbeiten an der Stationsatmosphäre und Ökonomisierungsprozessen im Krankenhauswesen.

### Ausflug auf die ‚Komfortstation‘. Von der unsichtbaren Arbeit an der Krankenhausatmosphäre

Während meiner Feldforschung bewegte ich mich in Begleitung von Reinigungs- und Pflegekräften eines Universitätsklinikums meist in Räumen, die im Stil der Krankenhausbauten der 1970er Jahre (Nickl 2014: 50) funktional und schmucklos gebaut waren und deren wenig ansprechende optische Wirkung im Alltag immer wieder Thema war. In Begleitung einer Mitarbeiterin der Hauswirtschaft bei einem Rundgang durch ein anderes Universitätsklinikum einer anderen Stadt lernte ich jedoch eine Komfortstation für Privatpatient_innen des Krankenhauses kennen. Ein Auszug aus meinen Feldnotizen:

Frau F. öffnete die Glastür zu einem Flur mit den Worten: „Und das ist unsere Komfortstation.“ Mir fällt sofort die andere Gestaltung des Flurs auf: An den Wänden hängen edel gerahmte Bilder abstrakter Kunst und an den Wänden sind dezente Farbeffekte durch Fußleisten u. a. zu sehen. […] Als wir das Zimmer betreten, bezieht eine Mitarbeiterin von Frau F. gerade das Bett. Das Zimmer ist schon farblich ganz anders gestaltet als die anderen Patient_innenzimmer: Die Möbel sind in kräftigen Farben gehalten, es gibt ein Ledersofa und einen Sessel und das Holz des Couchtischs findet sich als Vertäfelung hinter dem Bett wieder. […] Besonders beeindruckt bin ich von dem Badezimmer, das mit seinen runden Formen und dem strahlenden Weiß der Armaturen sehr edel wirkt. „Das ist ja wie im Hotel“, sage ich spontan und Frau F. sowie ihre Mitarbeiterin stimmen mir zu. (Feldnotizen, auch von Bose 2017: 134)

In meinen Eindrücken zeigt sich, was in neueren Artikeln zur Krankenhausarchitektur sowie Medienbeiträgen betont wird: Eine als angenehm empfundene Atmosphäre von Krankenhausräumen entsteht dann, wenn diese so wenig wie möglich an ein Krankenhaus erinnern. Der Architekt Hans Nickl betont, dass es bei der Raumgestaltung in Zukunft darum gehe, klinischen Räumen „einen hotelähnlichen Charakter“ (Nickl 2014: 60) zu verleihen, „in dem Komfort und Ambiente im Mittelpunkt stehen und eine Atmosphäre erzeugt wird, in der man sich wohlfühlt und die eben nicht an ein Krankenhaus erinnert“ (ebd.). In den Beschreibungen architektonischer Projekte^11^ wird deutlich, dass dafür genau an den Merkmalen angesetzt wird, die als für Krankenhäuser typisch gelten. Das Weiß und die Kargheit der Wände werden durch Farben und Kunstdrucke ersetzt; dem Geruch von Desinfektionsmitteln wird teils mit Aromatherapie entgegengesteuert (Behar et al. 2018). In der Argumentation architektonischer Gestaltung wird zugleich mit den spezifischen Anforderungen an „Räume des Gesundheitswesens“ argumentiert:

Abgesehen von funktionalen, technischen und hygienischen Bedürfnissen wächst der Anspruch der Menschen an umfassend gestaltete Räume im Gesundheitswesen mit hoher Aufenthaltsqualität und Wohlfühlatmosphäre und der Raum wird zum wesentlichen Faktor des Genesungsprozesses. (Nickl 2014: 60)

In diesem Zitat sowie in anderen Beiträgen der Architektur wird zwar auf die Relevanz der Hygiene hingewiesen, die durch architektonische Gestaltung unterstützt werden könne (Artlich et al. 2014: 71). Die alltägliche Arbeit jedoch, die den Gesamteindruck des Raumes und seine angenehme Atmosphäre aufrechterhält, sobald das architektonische Projekt umgesetzt wurde, wird nicht thematisiert.^12^ Das Weiß des Badezimmerinterieurs beispielsweise, das zu meinem positiven Gesamteindruck der Komfortstation beitrug, muss täglich geputzt werden, um diesen Eindruck zu gewährleisten. Hier kommt der Anspruch zum Tragen, den die Reinigungskraft Frau A. im Interview ausdrückte: Die Nutzer_innen der von ihr gereinigten Räume sollen sich wohlfühlen. Das Wohlfühlen lässt sich jedoch nicht lediglich durch eine aufwändige Gestaltung erreichen, sondern ist eine Frage des alltäglichen Bearbeitens und Instandhaltens. Doch diese Arbeit bleibt meist unsichtbar (auch von Bose 2017: 141).

Während auf der bereits beschriebenen Station eines anderen Klinikums darum gerungen wird, durch eine Grundreinigung und damit auch optische Aufhellung des Bodens hygienische Reinheit sowie den Eindruck hygienischer Reinheit herzustellen, der von einem Krankenhaus erwartet wird, ist die Komfortstation bereits mehrere Schritte weiter – soll hier die Assoziation mit einem Krankenhaus doch gänzlich ersetzt werden. Für unterschiedlich versicherte Patient_innen und angesichts des steigenden Wettbewerbs im Krankenhaussektor (Braun et al. 2010; Nickl 2014: 60) verweist die architektonische Gestaltung auf soziale Ungleichheiten im Gesundheitssystem. Klassenfragen an der Schnittstelle von Gesundheit und Krankheit schreiben sich damit auch in Räume und ihre Atmosphäre ein.

## Ausblick: Von der Arbeit an der Atmosphäre als Konfliktzone und der feminisierten Umweltfürsorge als Infektionsprävention

Leitfrage dieses Beitrags war, wie an der räumlichen Umwelt und, spezifischer, der Atmosphäre von Krankenhausräumen gearbeitet wird und von welchen Wissensordnungen diese Arbeit geprägt ist. Damit kamen unterschiedliche Akteur_innen von Reinigungspersonal bis hin zu Architekt_innen in den Blick, die an der räumlichen Umwelt im Krankenhaus mitarbeiten. Es wurde deutlich, dass diese Arbeit sowie die Bedeutung, die ihr jeweils beigemessen wird, nach wie vor vergeschlechtlicht ist und zum Gegenstand alltäglicher Aushandlungen wird.

Dies ist zunächst einmal auf das Wissen und die Debatten zurückzuführen, die hier zusammenlaufen. So wurde deutlich, dass die ganz alltägliche Umweltfürsorge in Krankenhäusern einerseits feminisiert und abgewertet ist, andererseits jedoch auch maßgeblich von Fragen der Hygiene geleitet wird. Sie ist zugleich durchdrungen und angetrieben von öffentlichen Debatten um Krankenhausinfektionen und ist mit changierenden Ansprüchen an Komfort, Ästhetik und Krankheitsprävention konfrontiert. Wird die Vorsicht und Sorge vor unsichtbaren Gefahren aktuell angesichts von Covid-19 auch im Alltag deutlich, durchzieht und bestimmt sie im Krankenhaus spätestens seit Aufkommen der Bakteriologie Ende des 19. Jahrhunderts die meisten Tätigkeiten. Die Unsichtbarkeit der Keime übersetzt sich nicht selten in die sichtbaren räumlichen Bedingungen sowie eine fühlbare Atmosphäre: Wird diese als sauber, ordentlich und in einem nicht ganz greifbaren Sinne als angenehm empfunden, wirkt sich dies auch positiv auf die Vermutung hygienischer Reinheit und damit auf das Vertrauen in die Gesundheitsversorgung aus. Unaufgeräumte oder schmutzig wirkende Räume und Gegenstände wiederum bestärken die Sorge vor unsichtbar lauernden Infektionsgefahren.

Ist die sprichwörtlich gewordene Krankenhausatmosphäre grundlegend negativ konnotiert, erscheint die Arbeit, eine vertrauenerweckende Atmosphäre herzustellen, besonders konfliktreich. Patient_innen setzen Erwartungen von Krankheitsprävention, Komfort und Service in Raumausstattung und -pflege, die von den dafür zuständigen Akteur_innen unter ihren Arbeitsbedingungen kaum zu leisten ist. Schmutz als hygienischer Gefahrenquelle kann bei Patient_innen Ekel- und Angstgefühle auslösen, bei Pflege- und Reinigungspersonal lassen sich angesichts nicht zu bewältigender Aufgabenberge Ärger und Erschöpfung beobachten.

Mit Blick auf die konkreten Räume werden Unterschiede in den Anforderungen deutlich, welche die Arbeit an der Atmosphäre jeweils hervorruft. Changiert das Bett zwischen einer schützenden und gefährdenden Intimität für unterschiedliche Akteur_innen, so ist das Krankenzimmer von Aushandlungen um Zuständigkeiten und um die Arbeit an einer Wohlfühlatmosphäre geprägt, die die Kapazitäten von Pflege als auch Reinigung übersteigt. Auf der Ebene der Station knüpfen sich an die vertrauensvolle Atmosphäre Fragen nach Ruf und Prestige der Klinik, für die Reinigungsarbeiten ein notwendiges, jedoch wenig beachtetes Mittel darstellen.

Das hier zu beobachtende *Cocooning*, die Fürsorge für die Umwelt der Patient_innen, ist von Diskursen um hygienische Reinheit und Reinlichkeit geprägt, deren Feminisierung und Rassifizierung eine lange historische Kontinuität aufweisen. In der Analyse werden sie zudem als Ausdruck und Effekt struktureller Bedingungen sichtbar: Dem Zeitdruck der Reinigungskräfte, der durch Ökonomisierungsprozesse wie der Politik des Outsourcings und damit Prekarisierung nicht-medizinischer Leistungen bedingt ist, dem Personalmangel in der Pflege und der nach wie vor vergeschlechtlichten Struktur der Krankenhaushierarchie, die den Status der jeweiligen Tätigkeiten markiert.

In Krankenhausräumen ist die Sorge für die Umwelt also immer zugleich auch eine Frage der Infektionsprävention. Somit verwundert es nicht erst seit der breiteren Debatte um „Systemrelevanz“, dass diese Arbeit sowie ihre Akteur_innen meist unsichtbar bleiben oder zumindest übersehen werden. Ein ethnografischer Einblick in Hygiene- und Sauberkeitspraktiken rückt diese Akteur_innen und ihre Arbeit ins Blickfeld. So wird deutlich, dass auch die Sorge für klinische Umwelten eingebettet ist in spezifische Macht- und Ungleichheitsverhältnisse, die sich bei genauerem Hinsehen als historisch gewachsen erweisen.

## Footnotes

1 Die folgenden Ausführungen zu historischen Hygiene- und Sauberkeitsdiskursen insbesondere für das Krankenhaus basieren auf von Bose (2017: 62–80).

2 Über rassistische Übergriffe und Diskriminierungen im Zusammenhang mit Covid-19 wurde unter anderem in folgenden Artikeln berichtet: Tagesschau (2020); Tagesspiegel (2020); SWR (2020).

3 Orte und Personen sind in diesem Beitrag anonymisiert und treten zunächst vor allem in ihrer beruflichen Funktion in Erscheinung, statt dass ihre soziale Positionierung entlang von Differenzkategorien wie *race, class, gender* ‚beschrieben‘ würde. Solche Markierungen werden dann vorgenommen, wenn sie für die Analyse wichtig erscheinen. Generell lässt sich aber sagen, dass hier eine Feminisierung und Ethnisierung von Carearbeit deutlich wird: Die Reinigungs- und Pflegekräfte, mit denen ich sprach, waren überwiegend Frauen und insbesondere die Reinigungskräfte wurden häufig migrantisch markiert. Für eine ausführliche Reflexion zu Repräsentationspraktiken in der ethnografischen Forschung vgl. von Bose (2019b), bezogen auf meine eigenen Rollen im Feld vgl. von Bose (2017: 17–18 und 84–89).

4 Im Krankenhausmanagement wird vor allem dann von Hotelleistungen gesprochen, wenn es um den nicht-klinischen Teil der Versorgung von Patient_innen geht, wie die Speiseversorgung, Reinigung/Hygiene und Textil‑/Wäscheversorgung (Behar et al. 2018: 208).

5 Zur Bedeutung weißer Reinheit und Weiblichkeit (Dietrich 2007).

6 Der weibliche Körper wiederum wird historisch sowohl mit Reinheit als auch Unreinheit und Schmutz assoziiert (Malich 2018: 120; Wolkowitz 2007: 18).

7 Dafür, so schreibt Susanne Kreutzer in ihrer Historie zur Reform der Krankenpflege, genoss der Beruf der Krankenschwester damals noch ein hohes Ansehen und großen Respekt (ebd.: 18) – ein Umstand, den heutige Pflegekräfte vermissen (von Bose 2017: 166).

8 Dass der letzte Satz in Vergangenheitsform formuliert ist („blieb ja alles an uns hängen“), verweist auf eine Neuerung zur Zeit des Interviews: Nun wurden vermehrt Servicekräfte beschäftigt, die eine Position zwischen Reinigung und Pflege einnehmen und Tätigkeiten wie Aufräumen und Essenabfrage ausführen. Diese stehen jedoch ebenfalls in der Kritik, da sie ebenso wenig zum „Stammpersonal“ gehören.

9 Eine Prioritätensetzung auf die medizinische Behandlung der Ärzt_innen, die Mehrarbeit für die Pflege bedeutet, lässt sich im Alltag vielfach beobachten. Eine in Eile abgezogene Infusion, die auf den Boden tropft, ein unordentlich hinterlassenes Behandlungszimmer, eine stehengelassene Kaffeetasse – all diese beiläufigen Gesten sind eingebettet in die entlang von Geschlechter- und Klassendifferenzen strukturierten Statushierarchien im Krankenhaus (vgl. auch von Bose 2017: 117–122).

10 Dies zeigen auch die Affekte, die mit der Aufgabenverteilung einhergehen können (das betretene, murmelnde Rechtfertigen der Pflegekräfte nach der ärztlichen Ermahnung zum Aufräumen oder die Empörung, die eine stehengelassene Kaffeetasse auslösen kann).

11 Für eine ausführlichere Analyse krankenhausarchitektonischer Projekte: von Bose (2017: 135–142).

12 Denkbar wäre etwa das Thematisieren von leicht zu reinigenden Materialien oder von Rückzugs- oder Arbeitsräumen für Reinigungs- und Hauswirtschaftspersonal.
